# Forced proximity promotes the formation of enduring cooperative relationships in vampire bats

**DOI:** 10.1098/rsbl.2022.0056

**Published:** 2022-04-06

**Authors:** Imran Razik, Bridget K. G. Brown, Gerald G. Carter

**Affiliations:** ^1^ The Ohio State University, Columbus, OH 43210, USA; ^2^ Smithsonian Tropical Research Institute, Balboa Ancón, Panama

**Keywords:** vampire bats, cooperation, social relationships, cooperative relationships, association

## Abstract

Spatial assortment can be both a cause and a consequence of cooperation. Proximity promotes cooperation when individuals preferentially help nearby partners, and conversely, cooperation drives proximity when individuals move towards more cooperative partners. However, these two causal directions are difficult to distinguish with observational data. Here, we experimentally test if forcing randomly selected pairs of equally familiar female common vampire bats (*Desmodus rotundus*) into close spatial proximity promotes the formation of enduring cooperative relationships. Over 114 days, we sampled 682 h of interactions among 21 females captured from three distant sites to track daily allogrooming rates over time. We compared these rates before, during and after a one-week period, during which we caged random triads of previously unfamiliar and unrelated vampire bats in proximity. After the week of proximity when all bats could again freely associate, the allogrooming rates of pairs forced into proximity increased more than those of the 126 control pairs. This work is the first to experimentally demonstrate the causal effect of repeated interactions on cooperative investments in vampire bats. Future work should determine the relative importance of mere association versus interactions (e.g. reciprocal allogrooming) in shaping social preferences.

## 1. Introduction

Spatial assortment can both be a cause and a consequence of cooperation. This principle, well understood from social evolution theory, applies to both ecological and evolutionary timescales, and across a great diversity of social life from microbes to humans [1–6]. Proximity drives cooperation when individuals primarily help nearby partners, and conversely, cooperation drives proximity when individuals move towards more cooperative partners. Although both forces are predicted by theory, these two causal directions are not easily distinguished with observational data.

Individuals forced to associate might form enduring cooperative relationships that persist beyond the period of forced proximity, similar to how randomly assigned first-year college roommates are more likely to be friends after graduating [7]. Several past results suggest that such a phenomenon occurs in common vampire bats (*Desmodus rotundus*), a species where preferred associations correlate with cooperative interactions, such as allogrooming and regurgitated food sharing [8–12]. Vampire bats housed together for months in captivity appeared to develop stronger social preferences for each other and then maintained these associations for at least 8 days after being released back into the wild [11]. Female pairs that were introduced to one another in small cages started food sharing relationships faster than pairs that first met in a larger cage with more options for social partners [12]. In field sites where vampire bats switch among roosts, pairwise roost-sharing rates predict allogrooming and food sharing [8,9,13]. However, all these studies lack the proper experimental controls to show causation.

Here, we experimentally tested if forcing proximity among randomly selected triads of female common vampire bats promotes the formation of enduring cooperative relationships. We measured allogrooming as a response because it is a cooperative and highly symmetrical investment of time and energy that is directed to individuals in need [14], sufficiently frequent during the formation of new relationships [12], and used by individuals to establish and maintain more costly food sharing relationships [12].

## 2. Material and methods

### (a) Study colony

Experimental subjects were 21 uniquely banded and captive female common vampire bats. Seven bats were sourced from each of three different sites in Panama (Lake Bayano, Tolé, or La Chorrera) that were 120–350 km apart and they were housed in an outdoor flight cage at the Smithsonian Tropical Research Institute in Gamboa, Panamá. See Razik *et al*. [15] and the electronic supplementary material for more details on the captive colony.

### (b) Experimental design

**Figure 1.**
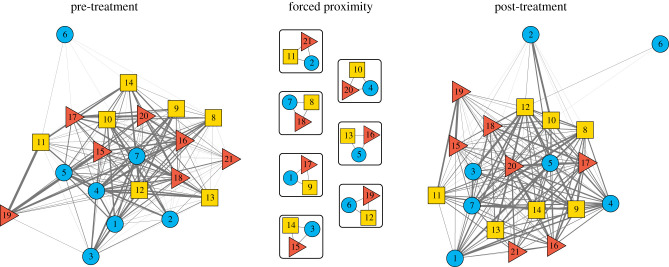
Experimental design. (*a*) Pre-treatment, (*b*) forced proximity and (*c*) post-treatment. Nodes are bats. Node colour/shape shows capture site (Lake Bayano = blue circles, Tolé = yellow squares, La Chorrera = red triangles). The widths of the grey links show the relative allogrooming log rates during the six-week pre-treatment phase, the one week of forced proximity and the nine-week post-treatment phase.

Subjects experienced three phases: pre-treatment (six weeks), forced proximity (one week) and post-treatment (nine weeks). During the pre-treatment phase, all bats could freely interact in a 2.1 × 1.7 × 2.3 m flight cage. During the treatment phase, we randomly assigned female bats into seven triads (including one bat from each site so that all individuals were previously unfamiliar), each housed in a 28 × 28 × 40 cm clear, acrylic observation cage. For the post-treatment phase, we released all bats back into the flight cage and monitored interactions (figure 1). We tracked grooming rates between all pairs and classified pairs into three types. We classified the 21 recently introduced pairs that were forced into proximity as ‘test dyads’, the other 126 recently introduced pairs as ‘control dyads’ and the 63 pairs of bats caught from the same site as ‘familiar dyads’.

To measure allogrooming, we used three infrared surveillance cameras (Foscam NVR Security System) to sample all social interactions among the uniquely banded bats for 3–6 h each day from 23 June 2019 to 14 October 2019. Over 114 days, we sampled 682 h of interactions, recording allogrooming bouts that were at least 5 s in duration, and noting both the actor and receiver. To measure dyadic allogrooming rates, we averaged the seconds of allogrooming per hour across all hours in which the actor and receiver could interact, first averaging the rates in both directions (most allogrooming is bidirectional), then transforming these values with *natural log* (*x* + 1), because the variance of allogrooming duration increases with the mean. We call these measures ‘allogrooming log rates’.

### (c) Statistical analyses

To test for an effect of forced proximity, we compared the mean change in allogrooming log rates from the pre-treatment to the post-treatment phase for test dyads, control dyads and familiar dyads, then calculated 95% confidence intervals (CI) for all mean changes using bootstrapping (percentile method in the *boot* R package) [16]. Using the same bootstrapping method, we calculated the 95% CI for the mean change in the proportion of total allogrooming log rates directed to familiar versus previously unfamiliar partners. To assess evidence of a trade-off between grooming familiar versus previously unfamiliar partners, we also tested for a negative correlation between the actor's mean change in grooming to these two partner types.

To test if the mean change in allogrooming log rates differed more than expected by chance between test and control groups, we used a permutation test. We compared the observed difference to a distribution of 5000 expected differences obtained from running the same analysis after randomly re-assigning bats from different capture sites into possible new forced-proximity triads. To check the robustness of our original result from this analysis, we also conducted several alternative analyses (see electronic supplementary material, information). Briefly, our results hold when (i) excluding 17 control dyads with pre-treatment allogrooming rates higher than the maximum rate observed in test dyads (to account for regression to the mean effects), (ii) removing two bats that were sampled fewer times than other females, (iii) removing bats that had a Staph infection during the pre-treatment period and (iv) drawing inferences from a mixed-effect model. All analyses confirm the original result of a clear difference between test and control groups.

To test if allogrooming log rates in the 21 test dyads during the forced-proximity phase predicted changes in allogrooming log rates between the pre-treatment and post-treatment phase, we fit a linear model with the change in allogrooming as a ranked response and forced-proximity allogrooming ranks as the effect. To test if forced-proximity allogrooming ranks predicted allogrooming during the post-treatment phase after controlling for baseline levels, we fit a linear model with post-treatment allogrooming rank as a response and both pre-treatment and forced-proximity allogrooming ranks as effects. Alongside parametric *p*-values, we present one-sided *p*-values from a permutation test (permutation-p) comparing the observed coefficients to those expected when allogrooming rates are randomized within each forced-proximity cage (5000 permutations).

## 3. Results

**Figure 2.**
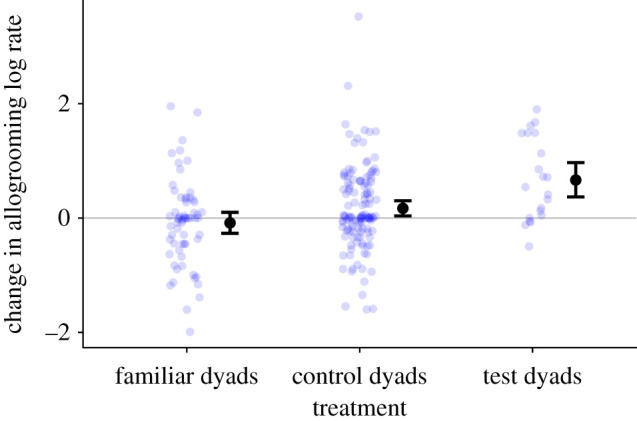
Changes in allogrooming in familiar, control and test dyads. Blue points are bat pairs. Larger black points are means with 95% CIs. Familiar dyads are from the same capture site. Test and control dyads are pairs of bats from different capture sites that were either forced into proximity or not, respectively.

We found that just one week of forced proximity between recently introduced and unrelated females increased their allogrooming rates, relative to a control group, after the manipulation ended. From the pre-treatment to the post-treatment phase, bats increased the proportion of their allogrooming directed to bats from different capture sites (mean increase = 0.06, 95% CI = [0.002, 0.11]). Allogrooming increased more in the test dyads that were forced into proximity compared to the control dyads (figure 2; mean increase for test dyads = 0.66 log s h^−1^ [0.37, 0.97], for control dyads = 0.17 log s h^−1^ [0.04, 0.30]; difference = 0.50 log s h^−1^, *p* = 0.002). This effect was evident throughout the post-treatment period (electronic supplementary material, figure S1). Although the bats formed new grooming relationships (figure 2), we found no evidence of a negative correlation between grooming directed to familiar versus relatively new partners (*r* = 0.25 [−0.21, 0.61], *n* = 21, *p* = 0.27).

Within the 21 test dyads, allogrooming rates during forced proximity were more clearly correlated with post-treatment rates (Spearman's *ρ* = 0.49, *p* = 0.025) than pre-treatment rates (*ρ* = 0.33, *p* = 0.15); however, we failed to find clear evidence that allogrooming ranks among the 21 test dyads during the forced proximity phase predicted changes in allogrooming (*β* = 0.28, *p* = 0.206, permutation test *p* = 0.15), or post-treatment allogrooming when controlling for the baseline pre-treatment allogrooming (*β* = 0.32, *p* = 0.096, permutation test *p* = 0.074; electronic supplementary material, figure S5).

## 4. Discussion

We introduced previously unfamiliar and unrelated vampire bats in captivity, allowing them to freely associate for six weeks in an outdoor flight cage, then forced randomly selected triads into proximity for one week. Over the next nine weeks, while bats could again freely associate, the allogrooming rates of pairs forced into proximity increased more than those of control pairs or previously unfamiliar pairs that were not forced into proximity. This finding shows that manipulating proximity can promote the formation of enduring cooperative relationships that persist beyond the manipulation period.

Although the bats formed new grooming relationships, we found no clear evidence that this required partner switching, which makes sense because the bats were removed from their wild social networks and presumably lost most of their original social ties. Animals capable of individual social recognition are likely to have a familiarity bias, leading to social preferences among individuals that happen to meet earlier in time or be closer in space. Early-life associations can be a cue for kin discrimination (e.g. [17]) and lead to strong preferences later in life [18–21]. For example, newborn pipistrelle bats (*Pipistrellus kuhlii*) raised in separate groups for six weeks preferred to associate with and groom familiar conspecifics and heterospecifics after being released into a common flight cage [20,21]. Preferred associations can also develop quickly during adulthood. For instance, guppies (*Poecilia reticulata*) develop new schooling preferences for familiar individuals over just 12 days, and the effect of familiarity appears stronger than that of phenotypic similarity [22,23].

The mechanism underlying the increased allogrooming remains unclear, but it was likely caused by some combination of elevated *association* (e.g. bats increased allogrooming with partners that were nearby more often) and *interaction* (e.g. bats increased allogrooming with partners that groomed them more). Although we failed to detect a clear correlation between allogrooming intensity within the forced-proximity period and changes in allogrooming afterwards, our power to detect this effect is far worse than our ability to detect the effect of forced proximity for three reasons: (i) we did not directly manipulate grooming, (ii) the forced-proximity grooming rates were based on far fewer observations (one week versus six/nine weeks) and (iii) we had far fewer dyads in the analysis (21 versus 147 dyads). Furthermore, past work on vampire bats strongly suggests that spatial proximity alone cannot entirely explain either the large variation in cooperative behaviour across individuals and pairs living in proximity in captivity [12,24] or the changes in cooperative relationships owing to manipulations of experience [12]. For example, allogrooming was a better predictor of new food sharing relationships within periods of forced proximity than within periods of free association [12]—the opposite of what is expected if food sharing is caused only by forced proximity. Decisions to help are therefore determined, at least in part, by partner experiences or traits. If individuals use frequent association as an honest cue of partner availability, and if availability is desirable in a social partner, then this could result in a positive feedback loop during the social bonding process, where bats preferentially groom frequent associates and preferentially associate with frequent groomers. Future experiments should disentangle the roles of interactions versus association in social bonding.

Given that social grooming or preening occurs across many species, tracking the emergence of these interactions among introduced strangers provides an opportunity to compare how social bonding varies across species. Many aspects of the social bonding process remain mysterious. If individuals that were forced to associate in the same small cage similarly perceived these shared experiences as negative, would this weaken or strengthen the process of social bonding? Animals might prefer partners with whom they share rewarding experiences because of simple associative learning; alternatively, negative or stressful experiences that are shared might facilitate or reinforce partner choice [25]. For instance, guppies that were exposed to an environment where they perceived high predation risk were found to exhibit stronger partner preferences for one another than those that were not [26]. Furthermore, to what extent is the process of social bonding influenced by the quantity and quality of other available partners? Vampire bats form new relationships faster in the absence of familiar partners [12], and the benefits of having fewer stronger bonds versus many weak ties could vary with many aspects of the social environment, such as social stability [24] and the number of alternative relationships. If social bonds require long-term cooperative investments, then do individuals have ‘investment strategies’ that depend on the marginal return on each additional unit of investment? Answering such questions will require controlling association while carefully manipulating interactions within pairs.

## Data Availability

Data and R code are available on Figshare: https://doi.org/10.6084/m9.figshare.19077431.v3 [27].
